# The U-shaped association between serum osmolality and 28-day mortality in patients with sepsis: a retrospective cohort study

**DOI:** 10.1007/s15010-024-02256-3

**Published:** 2024-04-22

**Authors:** Minghao Liang, Yifei Xu, Xiuhong Ren, Di Huang, Minyan Jin, Zhanjun Qiu

**Affiliations:** 1 College of Traditional Chinese Medicine, Shandong University of Traditional Chinese Medicine, Jinan, China; 2 First Clinical Medical College, Shandong University of Traditional Chinese Medicine, Jinan, China; 3 Qilu Hospital of Shandong University, Jinan, China; 4 The Affiliated Hospital of Shandong University of Traditional Chinese Medicine, Jinan, China

**Keywords:** Sepsis, Osmolality, Intensive care unit, Hypertonicity

## Abstract

**Background:**

Sepsis is a recognized global health challenge that places a considerable disease burden on countries. Although there has been some progress in the study of sepsis, the mortality rate of sepsis remains high. The relationship between serum osmolality and the prognosis of patients with sepsis is unclear.

**Method:**

Patients with sepsis who met the criteria in the Medical Information Mart for Intensive Care IV database were included in the study. Hazard ratios (HRs) and 95% confidence intervals (CIs) were determined using multivariable Cox regression. The relationship between serum osmolality and the 28-day mortality risk in patients with sepsis was investigated using curve fitting, and inflection points were calculated.

**Results:**

A total of 13,219 patients with sepsis were enrolled in the study; the mean age was 65.1 years, 56.9 % were male, and the 28-day mortality rate was 18.8 %. After adjusting for covariates, the risk of 28-day mortality was elevated by 99% (HR 1.99, 95%CI 1.74-2.28) in the highest quintile of serum osmolality (Q5 >303.21) and by 59% (HR 1.59, 95%CI 1.39-1.83) in the lowest quintile (Q1 ≤285.80), as compared to the reference quintile (Q3 291.38-296.29). The results of the curve fitting showed a U-shaped relationship between serum osmolality and the risk of 28-day mortality, with an inflection point of 286.9 mmol/L.

**Conclusion:**

There is a U-shaped relationship between serum osmolality and the 28-day mortality risk in patients with sepsis. Higher or lower serum osmolality is associated with an increased risk of mortality in patients with sepsis. Patients with sepsis have a lower risk of mortality when their osmolality is 285.80-296.29 mmol/L.

**Supplementary Information:**

The online version contains supplementary material available at 10.1007/s15010-024-02256-3.

## Introduction

Sepsis is a life-threatening multiorgan dysfunction caused by infection [1] and is a common disease in the intensive care unit (ICU). Sepsis is a recognized global health challenge that places a considerable disease burden on countries [2, 3]. A study has shown an incidence of sepsis of 437 per 100,000 person-years, with an in-hospital mortality rate of 17 % and a mortality rate of 26 % for severe sepsis [4]. Despite advances in the treatment of sepsis, with anti-infective and aggressive organ support for patients with sepsis, the mortality rate from sepsis remains high. The research has shown that early treatment of patients with sepsis can improve prognosis [1]. Therefore, early identification and treatment of the patient with poor prognosis of sepsis is crucial. This further emphasizes the importance of identifying risk factors associated with poor prognosis in sepsis.

Serum osmolality reflects the relative concentrations of solutes and water in the blood [5] and is an important indicator of the hydration status of the body [6]. Changes in the serum osmolality can produce osmotic pressure gradients that drive water transfer inside and outside the cell and play an important role in maintaining fluid balance, influencing cellular function, regulating electrolyte homeostasis, and regulating renal function [7–10]. The calculation of serum osmolality combines three indicators: sodium, glucose, and blood urea nitrogen (BUN). Studies have shown changes in blood glucose, sodium, and urea nitrogen during sepsis and have been noted to correlate with mortality risk [11–14]. It has been shown that serum osmolality levels correlate with short and long term outcomes in patients with acute heart failure [15]. A prospective cohort study demonstrated a nonlinear relationship between serum osmolality and all-cause and cardiovascular mortality in US adults [16]. In a cohort study that included 20,160 patients, it was shown that early high and low serum osmolality were independently associated with an increased risk of developing acute kidney injury (AKI) [17]. However, the relationship between serum osmolality and the prognosis in patients with sepsis is unclear. Therefore, we conducted a retrospective cohort study using the Medical Information Mart for Intensive Care Database IV (MIMIC-IV) database to investigate the relationship between serum osmolality levels and mortality risk in patients with sepsis in the ICU.

## Materials and methods

### Data source

This retrospective cohort study followed the guidelines in Strengthening the Reporting of Observational Studies in Epidemiology (STROBE) [18]. Participants for this study were extracted from MIMIC-IV 2.0. MIMIC-IV is an extensive publicly accessible database containing the information of all patients receiving care at Beth Israel Deaconess Medical Centre (Boston, Massachusetts, USA) from 2008 to 2019 [19]. It is an important database in the field of critical care. Minghao Liang, the first author of this study, attended the Protecting Human Research Participants training course and received access to the data (ID: 11506836). In order to protect the privacy of the participants, all their personal information was deleted. The Institutional Review Board of Beth Israel Deaconess Medical Centre approved this study, and exempt informed consent was granted.

### Study population

Sepsis was defined according to the Third International Consensus Definitions for Sepsis and Septic Shock (Sepsis-3) [1]. Patients with Sequential Organ Failure Assessment (SOFA) ≥2 and suspected or confirmed infection during hospitalization were included in the study [20]. We included only adult patients who were admitted to the ICU for the first time; patients with multiple ICU admissions or ICU stays <48 hours were excluded. Patients with missing osmolality-related data were excluded. We also excluded patients with end-stage renal disease and those on renal replacement therapy on the first day of ICU admission.

### Main variables and outcome variables

The main variable of the study was serum osmolality at the time of admission to the ICU. Simultaneous sodium, glucose, and BUN measurements were used to calculate serum osmolality [5, 21]. The formula for this calculation is: Na×2 + (glucose/18) + (BUN/2.8) [22]. Several serum osmolalities were calculated using this formula, and we used only the first data after admission to the ICU for the study. Follow-up time was from the start of ICU admission to the occurrence of the death event. The primary outcome of the study was 28-day mortality after ICU admission. In addition to this, we analyzed the relationship between serum osmolality and 90-day mortality.

### Variables

We extracted patients’ baseline information in the MIMIC-IV database. Basic information included age, sex, race, height, and weight. Vital signs included heart rate, saturation of peripheral oxygen (SPO_2_), temperature, systolic blood pressure (SBP), diastolic blood pressure (DBP), and urine output (UO). Laboratory tests included sodium, glucose, BUN, bicarbonate, calcium, white blood cells (WBC), platelet, hemoglobin, serum creatinine (SCr), and PH. Scoring data included SOFA score, Charlson comorbidity index (CCI), and acute physiology score III (APS III). Comorbidity information included chronic lung disease, congestive heart failure, AKI, and diabetes mellitus. Interventions included the use of ventilator and the use of loop diuretics. All of the above information uses the first data within 24 hours of admission to the ICU. If there is more than one result within 24 hours, the first data is selected.

### Statistical analysis

The data were classified into 5 groups based on quintiles of serum osmolality (Q1 ≤285.80 mmol/L, Q2 285.80-291.38 mmol/L, Q3 291.38-296.29 mmol/L, Q4 296.29-303.21 mmol/L, Q5 >303.21 mmol/L). In the analysis of baseline information, categorical variables were described as percentages, and continuous variables were expressed as mean ± standard deviation or median (interquartile range). Chi-square test was used to describe differences between groups for categorical variables. Continuous variables with normal distribution were compared using one-way ANOVA. Continuous variables with skewed distributions were compared using the Kruskal−Wallis H test. Univariate Cox regression was used to assess the relationship between prognostic factors and the 28-day mortality risk. Multivariable Cox regression was used to assess the relationship between serum osmolality and 28-day mortality. Based on the results of univariate regression and clinical experience, we constructed three models to adjust for confounders. Model 1 was adjusted for sex, age, ethnicity, height, and weight. Model 2 was further adjusted for heart rate, SPO_2_, temperature, SBP, DBP, UO, bicarbonate, calcium, WBC, platelets, hemoglobin, SCr, and PH. Model 3 was further adjusted for AKI, diabetes mellitus, congestive heart failure, chronic pulmonary disease, ventilator use, and loop diuretics use. We analyzed 90-day mortality using the same methods. The relationship between serum osmolality and 28-day mortality in patients with sepsis was described using multivariate-adjusted restricted cubic spline. The recursive algorithm was used to calculate the inflection point, and a two-piecewise Cox proportional hazard regression model including values on both sides of the inflection point was constructed to assess the threshold effect of serum osmolality on 28-day mortality. In sensitivity analyses, we stratified participants according to age (≤65, >65), sex (female, male), AKI (yes, no), and diabetes mellitus (yes, no) and performed curve fitting to assess the stability of the results. We performed stratified curve fitting depending on the origin of the patients and whether they were diagnosed with septic shock. We also extracted the amount of fluid the patients received on the first day after admission to the ICU and examined the distribution of fluid volumes in osmolality subgroups. We divided the amount of fluid received equally into 3 groups and performed curve fitting of serum osmolality to 28-day mortality risk in each of these 3 groups. Finally we examined the relationship between serum osmolality and the mortality risk at different sites of infection. The covariates have missing values < 30%, and we use multiple interpolations to deal with the missing values. The data were analyzed using R software (version 4.2.2) and Free Statistical software (version 1.8). *P*<0.05 was considered significant.

## Results

### Study population and baseline characteristics

**Fig. 1:**
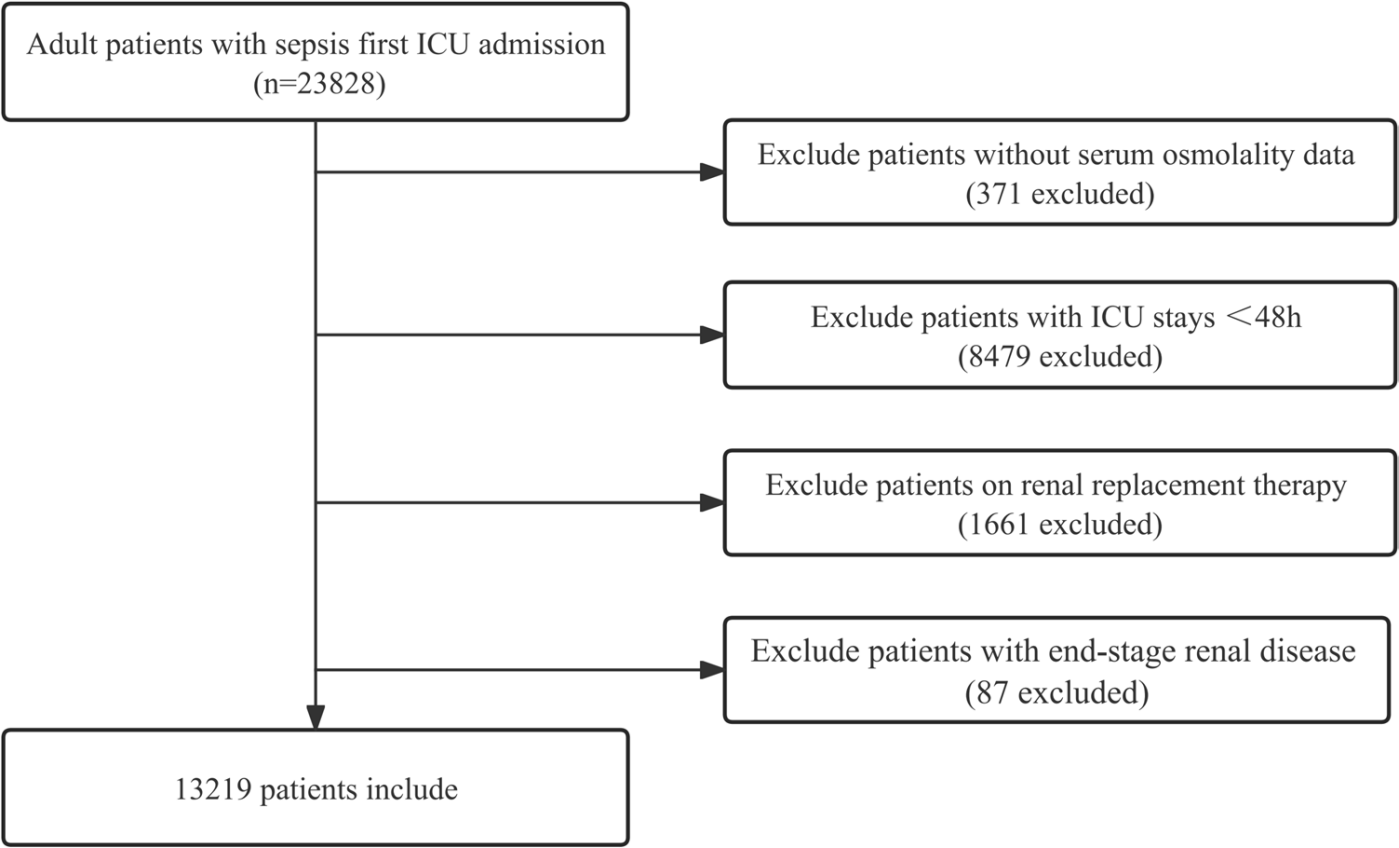
Flowchart of study patients

We recruited 23,828 patients who met the diagnostic criteria for sepsis and were first admitted to the ICU in the MIMIC-IV database. After excluding patients with missing osmolality data, ICU admissions <48h, use of renal replacement therapy, and end-stage renal disease, 13,219 participants were enrolled in the study (Fig. 1). Table 1 shows the baseline characteristics of participants grouped by serum osmolality levels. The mean age of all participants was 65.1±16.6 years, of which 56.9% were male and 64.0% were white. There were 27.0% of participants with chronic pulmonary disease and 28.5% of participants with diabetes mellitus. The 28-day mortality rate was 18.8%, and the 90-day mortality rate was 25.1%. Patients with lower osmolality had faster heart rates and higher levels of platelets. Patients with higher osmolality were older, had a smaller proportion of whites, had lower body temperatures, and had lower UO.

**Table 1:** Baseline characteristics of participants according to osmolality levels

Variables	Total (*n* = 13219)	Baseline serum osmolality levels (mmol/L)					*P*-value
		Q1	Q2	Q3	Q4	Q5	
		≤ 285.80	285.80–291.38	291.38–296.29	296.29–303.21	> 303.21	
		(*n* = 2644)	(*n* = 2642)	(*n* = 2642)	(*n* = 2647)	(n = 2644)	
Age (year)	65.1 ± 16.6	60.6 ± 17.0	62.9 ± 16.9	64.7 ± 16.5	67.2 ± 15.7	69.9 ± 15.2	< 0.001
Male (%)	7518 (56.9)	1479 (55.9)	1506 (57)	1531 (57.9)	1517 (57.3)	1485 (56.2)	0.566
Ethnicity, white (%)	8457 (64.0)	1690 (63.9)	1713 (64.8)	1731 (65.5)	1704 (64.4)	1619 (61.2)	0.015
Height (cm)	169.0 ± 10.6	169.1 ± 10.4	169.2 ± 10.7	169.5 ± 10.7	169.2 ± 10.7	168.0 ± 10.7	< 0.001
Weight (kg)	82.2 ± 23.9	79.2 ± 22.8	82.4 ± 24.3	83.7 ± 23.9	83.7 ± 23.2	81.7 ± 25.1	< 0.001
Vital signs							
Heart rate (bpm)	90.7 ± 20.7	94.0 ± 21.5	89.6 ± 19.8	88.3 ± 20.4	90.0 ± 20.5	91.7 ± 21.1	< 0.001
SPO_2_ (%)	97.1 ± 4.1	96.9 ± 3.8	97.2 ± 4.1	97.5 ± 3.7	97.0 ± 4.2	96.7 ± 4.4	< 0.001
Temperature (℉)	98.1 ± 3.6	98.4 ± 3.0	98.2 ± 3.3	98.1 ± 2.5	98.0 ± 3.9	97.9 ± 4.8	< 0.001
SBP (mmHg)	120.2 ± 25.2	119.2 ± 24.9	119.0 ± 24.8	119.5 ± 24.7	121.1 ± 25.9	122.0 ± 25.5	< 0.001
DBP (mmHg)	67.7 ± 18.9	68.3 ± 17.9	67.7 ± 18.8	67.2 ± 19.2	67.7 ± 19.2	67.8 ± 19.4	0.344
UO (ml)	1613.0 (1035.0, 2435.0)	1728.5 (1079.0, 2565.0)	1690.0 (1171.0, 2485.0)	1660.0 (1088.2, 2435.0)	1585.0 (1015.0, 2400.0)	1408.5 (868.2, 2236.2)	< 0.001
Laboratory results							
Sodium (mmol/L)	138.7 ± 5.6	132.6 ± 5.1	137.4 ± 2.4	139.2 ± 2.8	140.8 ± 2.9	143.7 ± 6.1	< 0.001
Potassium (mmol/L)	4.2 ± 0.7	4.2 ± 0.8	4.2 ± 0.7	4.1 ± 0.7	4.2 ± 0.7	4.2 ± 0.8	< 0.001
Glucose (mg/dL)	130.0 (108.0, 167.0)	117.0 (100.0, 143.0)	124.0 (104.0, 151.0)	130.0 (109.0, 160.0)	138.0 (114.0, 178.0)	157.0 (118.0, 222.0)	< 0.001
BUN (mg/dL)	19.0 (13.0, 31.0)	14.0 (10.0, 20.0)	16.0 (12.0, 22.0)	18.0 (13.0, 25.0)	22.0 (16.0, 32.0)	41.0 (25.0, 62.0)	< 0.001
Bicarbonate (mEq/L)	22.6 ± 4.7	22.5 ± 4.2	22.8 ± 4.1	23.0 ± 4.2	22.6 ± 4.7	22.1 ± 6.0	< 0.001
Calcium (mg/dL)	8.2 ± 0.9	8.1 ± 0.8	8.2 ± 0.8	8.2 ± 0.8	8.2 ± 1.0	8.3 ± 1.1	< 0.001
WBC (k/uL)	11.9 (8.4, 16.4)	11.4 (8.0, 16.0)	11.9 (8.6, 16.0)	11.9 (8.5, 16.2)	11.9 (8.7, 16.5)	12.3 (8.5, 17.4)	< 0.001
Platelet (k/uL)	186.0 (132.0, 255.0)	192.0 (132.0, 274.2)	190.0 (135.0, 255.0)	181.0 (129.2, 243.0)	184.0 (132.0, 244.0)	186.0 (129.8, 262.0)	< 0.001
Hemoglobin (g/dL)	10.7 ± 2.3	10.5 ± 2.2	10.8 ± 2.2	10.9 ± 2.2	10.8 ± 2.3	10.4 ± 2.2	< 0.001
SCr (mg/dL)	1.0 (0.7, 1.4)	0.8 (0.6, 1.1)	0.9 (0.7, 1.1)	0.9 (0.7, 1.2)	1.0 (0.8, 1.4)	1.5 (1.0, 2.3)	< 0.001
PH	7.4 ± 0.1	7.4 ± 0.1	7.4 ± 0.1	7.4 ± 0.1	7.4 ± 0.1	7.3 ± 0.1	< 0.001
Score system, points							
SOFA score	3.5 ± 1.9	3.3 ± 1.7	3.3 ± 1.7	3.4 ± 1.8	3.6 ± 1.8	4.0 ± 2.2	< 0.001
CCI	5.7 ± 2.9	5.2 ± 3.0	5.2 ± 2.8	5.4 ± 2.7	6.0 ± 2.9	6.8 ± 2.9	< 0.001
APSIII	56.4 ± 24.0	54.4 ± 23.7	50.5 ± 22.1	52.0 ± 22.7	56.7 ± 23.1	68.1 ± 24.4	< 0.001
Comorbidity disease							
Chronic pulmonary disease (%)	3568 (27.0)	658 (24.9)	669 (25.3)	681 (25.8)	785 (29.7)	775 (29.3)	< 0.001
Congestive heart failure (%)	3952 (29.9)	589 (22.3)	652 (24.7)	696 (26.3)	891 (33.7)	1124 (42.5)	< 0.001
AKI (%)	7382 (55.8)	1326 (50.2)	1364 (51.6)	1492 (56.5)	1578 (59.6)	1622 (61.3)	< 0.001
Diabetes mellitus (%)	3773 (28.5)	482 (18.2)	642 (24.3)	688 (26)	860 (32.5)	1101 (41.6)	< 0.001
Interventions							
Ventilator use (%)	10361 (78.4)	1994 (75.4)	2090 (79.1)	2132 (80.7)	2108 (79.6)	2037 (77)	< 0.001
Loop diuretics use(%)	1123 (8.5)	210 (7.9)	235 (8.9)	214 (8.1)	205 (7.7)	259 (9.8)	0.044
Osmolality (mmol/L)	295.0 ± 14.3	278.3 ± 8.9	288.7 ± 1.6	293.8 ± 1.4	299.5 ± 2.0	314.9 ± 14.3	< 0.001
28-day mortality (%)	2482 (18.8)	509 (19.3)	338 (12.8)	340 (12.9)	499 (18.9)	796 (30.1)	< 0.001
90-day mortality (%)	3322 (25.1)	681 (25.8)	482 (18.2)	485 (18.4)	655 (24.7)	1019 (38.5)	< 0.001

*SPO*_*2*_ saturation of peripheral oxygen, *SBP* systolic blood pressure, *DBP* diastolic blood pressure, *UO* urine output, *BUN* blood urea nitrogen, *WBC* white blood count, *SCr* serum creatinine, *SOFA score* sequential organ failure assessment score, *CCI* Charlson comorbidity index, *APS* III acute physiology score III, *AKI* acute kidney injury

### Multivariable Cox regression analysis

After performing univariate Cox regression analyses (Supplementary Table S1), we constructed three multivariable Cox regression models to assess the relationship between serum osmolality and the 28-day risk of mortality in patients with sepsis. Table 2 details the relationship between serum osmolality and the 28-day mortality risk, with effect values expressed as hazard ratios (HRs) and 95% confidence intervals (CIs). In the unadjusted model, the risk of mortality was increased by 55% (HR 1.55, 95%CI 1.35-1.78) in Q1 when compared with osmolality in Q3, and the risk of mortality was elevated by 162% (HR 2.62, 95%CI 2.30-2.97) in the highest quintile Q5. In model 3 (adjusted for sex, age, ethnicity, height, weight, heart rate, SPO_2_, temperature, SBP, DBP, UO, bicarbonate, calcium, WBC, platelets, hemoglobin, SCr, PH, AKI, diabetes mellitus, congestive heart failure, chronic pulmonary disease, ventilator use, and loop diuretics use), using Q3 as the reference, the risk of mortality was elevated by 59% (HR 1.59, 95%CI 1.39-1.83) in Q1 and 99% (HR 1.99, 95%CI 1.74-2.28) in Q5. We observed similar results in the relationship between serum osmolality and 90-day mortality risk in patients with sepsis (Supplementary Table S2). In model 3, as compared to Q3, the 90-day risk of mortality was increased by 51% (HR 1.51, 95%CI 1.35-1.70) and 79% (HR 1.79, 95%CI 1.59-2.00) for Q1 and Q5, respectively.

**Table 2:** Association of osmolality levels with 28-day mortality in patients with sepsis

Variable	Crude Model		Model 1		Model 2		Model 3	
	HR 95% CI	*P* value	HR 95% CI	*P* value	HR 95% CI	*P* value	HR 95% CI	*P* value
Q1 ≤285.80	1.55 (1.35–1.78)	<0.001	1.62 (1.41–1.86)	<0.001	1.61 (1.4–1.85)	<0.001	1.59 (1.39–1.83)	<0.001
Q2 (285.80-291.38)	0.99 (0.85–1.16)	0.936	1.02 (0.87–1.18)	0.842	1.02 (0.88–1.19)	0.773	1.02 (0.88–1.19)	0.767
Q3 (291.38-296.29)	1(Ref)		1(Ref)		1(Ref)		1(Ref)	
Q4 (296.29-303.21)	1.52 (1.32–1.74)	<0.001	1.45 (1.26–1.66)	<0.001	1.36 (1.18–1.56)	<0.001	1.37 (1.19–1.57)	<0.001
Q5 >303.21	2.62 (2.30–2.97)	<0.001	2.30 (2.03–2.62)	<0.001	1.95 (1.7–2.22)	<0.001	1.99 (1.74–2.28)	<0.001
P for trend		<0.001		<0.001		<0.001		<0.001

Crude model adjusted for none

Model 1 adjusted for age, sex, ethnicity, height, and weight

Model 2 adjusted for Model 1+heart rate, SPO_2_, temperature, SBP, DBP, UO, bicarbonate, calcium, WBC, PLT, hemoglobin, SCr, and PH

Model 3 adjusted for Model 2+ AKI, diabetes mellitus, congestive heart failure, chronic pulmonary disease, ventilator use, and loop diuretics use

*HR* hazard ratio, *CI* confidence interval, *Ref* reference

We observed a lower and similar risk of mortality in Q2 and Q3 of osmolarity. Therefore, we combined Q2 and Q3, and used Q2+Q3 as a reference for another multivariable Cox regression analysis. After adjusting for the covariates in model 3 in Table 3 we found that there was a 57% (HR 1.57, 95%CI 1.40-1.77) higher risk of mortality at 28 days in Q1 compared to Q2+Q3, and a 97% (HR 1.97, 95%CI 1.76-2.20) higher in the Q5. We observed similar results in the 90-day risk of mortality (Supplementary Table S3). The risk of mortality was lower at osmolarity of 285.80-296.29 mmol/L.

**Table 3:** Association of osmolality levels with 28-day mortality in patients with sepsis after combining Q2 and Q3

Variable	Crude Model		Model 1		Model 2		Model 3	
	HR 95% CI	*P* value	HR 95% CI	*P* value	HR 95% CI	*P* value	HR 95% CI	*P* value
Q1 ≤ 285.80	1.55 (1.39~1.74)	<0.001	1.61 (1.43~1.8)	<0.001	1.59 (1.42~1.79)	<0.001	1.57 (1.40~1.77)	<0.001
Q2+Q3 (285.80-296.29)	1(Ref)		1(Ref)		1(Ref)		1(Ref)	
Q4 (296.29-303.21)	1.52 (1.35~1.71)	<0.001	1.44 (1.28~1.61)	<0.001	1.34 (1.2~1.51)	<0.001	1.35 (1.2~1.52)	<0.001
Q5 >303.21	2.62 (2.37~2.91)	<0.001	2.29 (2.06~2.53)	<0.001	1.92 (1.72~2.15)	<0.001	1.97 (1.76~2.20)	<0.001
Trend.test		<0.001		<0.001		<0.001		<0.001

Crude model adjusted for none

Model 1 adjusted for age, sex, ethnicity, height, and weight

Model 2 adjusted for Model 1+heart rate, SPO_2_, temperature, SBP, DBP, UO, bicarbonate, calcium, WBC, PLT, hemoglobin, SCr, and PH

Model 3 adjusted for Model 2+ AKI, diabetes mellitus, congestive heart failure, chronic pulmonary disease, ventilator use, and loop diuretics use

*HR* hazard ratio, *CI* confidence interval, *Ref* reference

### Analyses of the U shape association

**Fig. 2:**
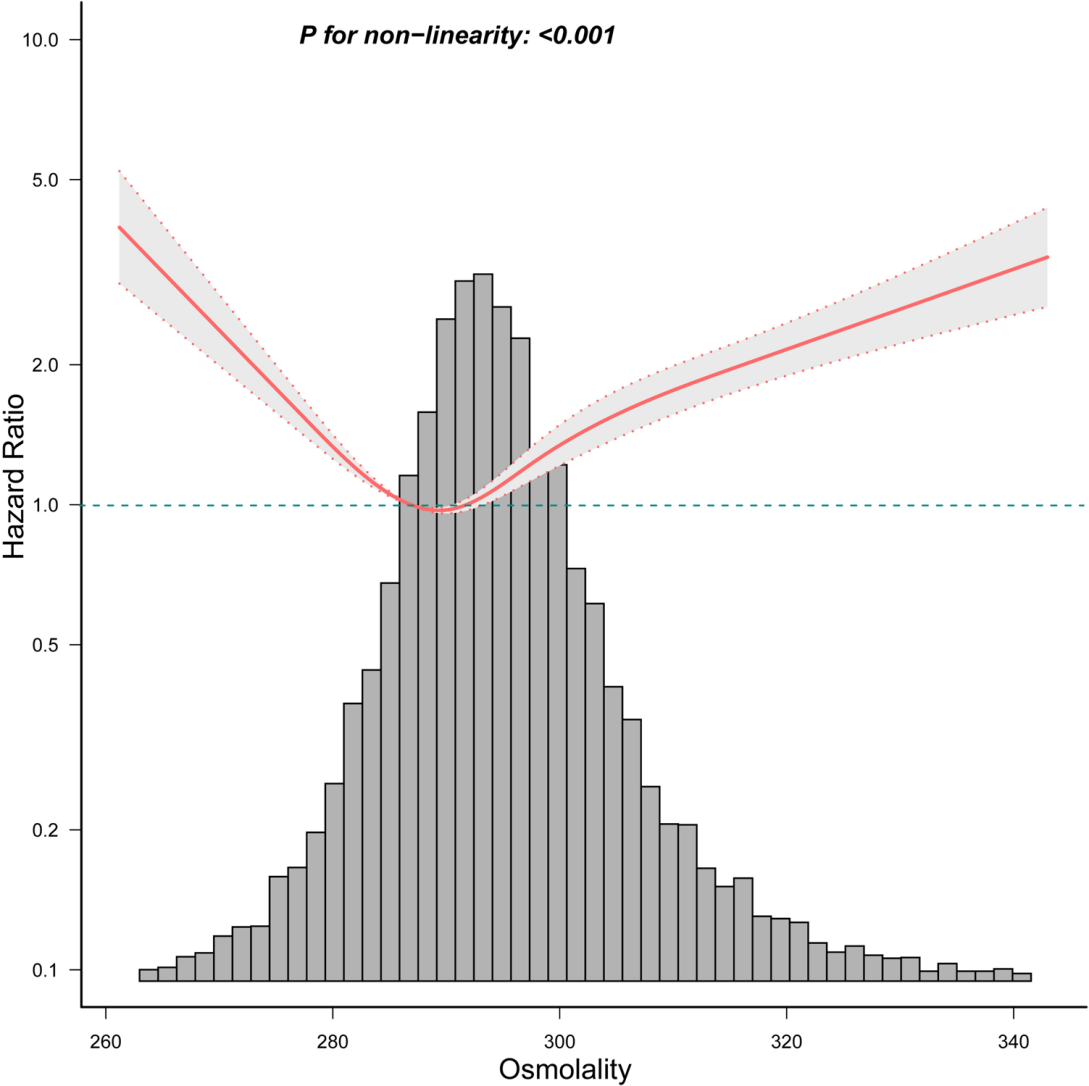
Relationship between osmolality and 28-day mortality

After adjusting for covariates in model 3, we found a U-shaped relationship between serum osmolality and 28-day mortality in curve fitting (*P* for non-linearity<0.001, Fig. 2). We also found this U-shaped relationship between osmolality and 90-day mortality risk (Supplementary Figure S1). We found an inflection point at 286.9 mmol/L (Table 4). When osmolality <286.9 mmol/L, the 28-day mortality risk decreased by 4.5% (HR 0.955, 95% CI 0.941-0.969) for every 1 mmol/L increase. At osmolality ≥286.9 mmol/L, there was a 2.8% (HR 1.028, 95% CI 1.023-1.032) increase in the 28-day mortality risk for every 1 mmol/L increase in osmolality.

**Table 4:** Threshold effect analysis of the relationship between osmolality and 28-day mortality of patients with sepsis

Threshold of osmolality	HR	95% CI	*P* value
< 286.9	0.955	0.941~0.969	< 0.001
≥ 286.9	1.028	1.023~1.032	< 0.001
Nonlinear test			<0.001

The data were adjusted for all the covariates of Model 3.

### Sensitivity analysis

To verify the stability of the U-shaped relationship between serum osmolality and 28-day mortality, we performed curve fitting stratified by age, sex, AKI, and diabetes, respectively. The results of the stratified curve fitting were shown in Supplementary Figure S2, and this U-shaped relationship remained stable across subgroups. In our study 47.2% of the patients were from the emergency room, 28.5% from the hospital and 24.2% from other sources. The U-shaped relationship between serum osmolality and the risk of mortality was still found in the curve fitting for the three sources (Supplementary Figure S3). We had a similar finding in septic shock (Supplementary Figure S4). The median amount of fluid received by all patients on the first day was 3343.9 ml (Supplementary Table S4). The amount of fluid received on the first day was not statistically different across osmolality subgroups (P>0.05). The results of stratified curve fitting showed that the U-shaped relationship between serum osmolality and 28-day mortality risk remained stable across these three subgroups (Supplementary Figure S5). The same relationship was observed in the 90-day mortality risk. We found that 54.1% of the patients had the site of infection in the lungs and 19.9% in the urinary tract (Supplementary Table S5). This U-shaped relationship remained stable across sites of infection (Supplementary Figure S6).

## Discussion

This study investigated the relationship between serum osmolality and mortality risk in patients with sepsis. After adjusting for relevant confounders, we found a U-shaped relationship between serum osmolality and mortality risk at 28 and 90 days in patients with sepsis. Lower or higher osmolality levels are associated with an increased risk of mortality. The 28-day risk of mortality decreased by 4.5% for every 1 mmol/L increase in osmolality when osmolality <286.9 mmol/L. When osmolality >286.9 mmol/L, each 1 mmol/L increase in osmolality was associated with a 2.8% increase in the 28-day risk of mortality. The risk of mortality was lower at osmolarity of 285.80-296.29 mmol/L. Serum osmolality should be of interest in identifying sepsis patients at high risk of mortality because of its ease of calculation and low cost.

The serum osmolality is the concentration of all solutes in serum and is important for maintaining stable cell volume [23]. Changes in the osmolality can create osmotic pressure gradients that promote water transfer. In hypotonic conditions, cells swell as they gain water, and in hypertonic conditions, they shrink as they lose water, both affecting normal physiological cell function [24]. It has been shown that low serum osmolality independently predicts mortality and readmission rates after discharge in patients with heart failure, whereas these associations were not observed in patients with high osmolality [25]. In the general population, serum osmolality has been observed to correlate nonlinearly with all-cause mortality and cardiovascular mortality [16]. Reduced lung function is also thought to be associated with increased osmolality [26]. In critically ill patients, serum osmolality is also an important indicator of some clinical outcomes. High serum osmolality and low serum osmolality are considered to be independently associated with an increased risk of developing AKI [17]. High serum osmolality is also thought to be associated with increased mortality from cardiac, cerebral, vascular, and gastrointestinal admission diagnoses [27].

Although the mechanism between serum osmolality and the risk of mortality in patients with sepsis is not clear, the current findings may give some explanation. It has been shown that hyperosmolality can lead to an increase in intracellular Ca^2+^ and reactive oxygen species concentrations, promoting endoplasmic reticulum stress and leading to cardiomyocyte apoptosis [28, 29]. The kidney plays an important role in osmolarity regulation and is most susceptible to osmotic stress. A study has shown that hyperosmolarity has a toxic effect on renal tubular epithelial cells, inducing oxidative stress and cytoskeletal damage, which in turn leads to kidney injury [30, 31]. In the presence of high osmolarity, the function of the blood-brain barrier is disrupted, resulting in an increase in cytokines, chemokines, and cell adhesion molecules [32, 33]. This shows that a state of high osmolality is associated with multiple organ impairment, which is consistent with the definition of multiple organ dysfunction in sepsis. However, the relationship between low serum osmolality and increased risk of mortality is unclear, it is possible that it is related to overhydration [34], but this needs to be demonstrated in further studies.

Our study has the following advantages. First, we are the first to study the relationship between serum osmolality and the risk of mortality in patients with sepsis. Second, we found a U-shaped relationship between serum osmolality and the risk of mortality at 28-day and 90-day using curve fitting. Third, we performed sensitivity analyses and curve fitting between different subgroups to verify the stability of the U-shaped relationship. Some disadvantages have to be considered. First, this was a retrospective study unable to make causal inferences, but we adjusted for potential confounders to ensure stability of results. Second, this is a single-center study, and future multi-center prospective studies are needed to validate this conclusion. Third, as the patients originated from different places, we were unable to access some of the factors that have an impact on osmolality before admission to the ICU, such as the amount of fluid received. However, we did our best to extract the amount of fluid in the 24h after patients were admitted to the ICU. The analyses showed no significant association between the amount of fluid received and osmolality. Curve fitting of the low, medium, and high subgroups based on the amount of fluid received on the first day showed that there was still a U-shaped relationship between serum osmolality and the mortality risk. This suggests that the amount of fluid received had no significant effect on this U-shaped relationship. Despite these disadvantages, our study of the relationship between serum osmolality and the risk of mortality in patients with sepsis remains of interest.

## Conclusion

There is a U-shaped relationship between serum osmolality and risk of mortality in patients with sepsis. Increases or decreases in serum osmolality levels are associated with mortality risk. Patients with sepsis have a lower risk of mortality when their osmolality is 285.80-296.29 mmol/L. Serum osmolality levels can be used to stratify the management of patients with sepsis.

## Supplementary Information

Below is the link to the electronic supplementary material.

Supplementary file1 (DOCX 166995 KB)

## Data Availability

This study uses publicly available databases, and all data are available at https://physionet.org/content/mimiciv.
