# Supplementary material for: The U-shaped association between serum osmolality and 28-day mortality in patients with sepsis: a retrospective cohort study
Source: Infection. 2024 Apr 22;52(5):1931–9. doi: 10.1007/s15010-024-02256-3 (PMC11499401; doi:10.1007/s15010-024-02256-3)
Supplement: Supplementary file 1 — Supplementary file1 (DOCX 166995 KB) [file 15010_2024_2256_MOESM1_ESM.docx]

**Table S1 Results of univariate analysis of 28-day mortality.**

| **Characteristic** | **HR 95% CI** | ***P-value*** |
| --- | --- | --- |
| Age | 1.03 (1.02-1.03) | < 0.001 |
| Male | 0.89 (0.83-0.97) | 0.006 |
| Ethnicity | 0.81 (0.75-0.88) | < 0.001 |
| Height | 0.99 (0.98-0.99) | < 0.001 |
| Weight | 0.99 (0.99-0.99) | < 0.001 |
| Heart rate | 1.0061 (1.0042-1.0079) | < 0.001 |
| SPO_2_ | 0.97 (0.96-0.98) | < 0.001 |
| Temperature | 0.98 (0.98-0.99) | < 0.001 |
| SBP | 1.0011 (0.9996-1.0027) | 0.154 |
| DBP | 1.0031 (1.0011-1.0051) | 0.003 |
| UO | 0.9997 (0.9996-0.9997) | < 0.001 |
| Sodium | 0.9962 (0.9891-1.0034) | 0.306 |
| Glucose | 1.02 (1.01-1.03) | < 0.001 |
| BUN | 1.04 (1.04-1.04) | < 0.001 |
| Bicarbonate | 0.97 (0.96-0.98) | < 0.001 |
| Calcium | 0.97 (0.93-1.01) | 0.146 |
| WBC | 1.0074 (1.0053-1.0095) | < 0.001 |
| Platelet | 1.0006 (1.0002-1.0009) | 0.001 |
| Hemoglobin | 0.98 (0.96-1) | 0.015 |
| Scr | 1.19 (1.16-1.23) | < 0.001 |
| PH | 0.19 (0.13-0.27) | < 0.001 |
| SOFA score | 1.1 (1.08-1.12) | < 0.001 |
| CCI | 1.19 (1.17-1.2) | < 0.001 |
| APS III | 1.03 (1.03-1.03) | < 0.001 |
| Chronic pulmonary disease | 1.11 (1.02-1.21) | 0.02 |
| Congestive heart failure | 1.29 (1.19-1.41) | < 0.001 |
| AKI | 1.54 (1.42-1.67) | < 0.001 |
| Diabetes mellitus | 0.94 (0.86-1.03) | 0.177 |
| Ventilator use | 0.92 (0.84-1.01) | 0.073 |
| Loop diuretics use | 1.29 (1.13-1.47) | < 0.001 |

SPO_2_, saturation of peripheral oxygen; SBP, systolic blood pressure; DBP, diastolic blood pressure; UO, urine output; BUN, blood urea nitrogen; WBC, white blood count, SCr, serum creatinine; SOFA score, sequential organ failure assessment score; CCI, Charlson comorbidity index; APS III, acute physiology score III; AKI, acute kidney injury.

**Table S2 Association of osmolality levels with 90-day mortality in patients with sepsis.**

| **Variable** | **Crude Model** | | **Model 1** | | **Model 2** | | **Model 3** | |
| --- | --- | --- | --- | --- | --- | --- | --- | --- |
|  | **HR 95% CI** | ***P*-value** | **HR 95% CI** | ***P*-value** | **HR 95% CI** | ***P*-value** | **HR 95% CI** | ***P*-value** |
| Q1 ≤285.80 | 1.47 (1.31~1.66) | <0.001 | 1.55 (1.38~1.74) | <0.001 | 1.53 (1.36~1.73) | <0.001 | 1.51 (1.35~1.70) | <0.001 |
| Q2 (285.80-291.38) | 0.99 (0.88~1.13) | 0.913 | 1.02 (0.9~1.15) | 0.802 | 1.02 (0.9~1.16) | 0.724 | 1.02 (0.9~1.16) | 0.76 |
| Q3 (291.38-296.29) | 1(Ref) |  | 1(Ref) |  | 1(Ref) |  | 1(Ref) |  |
| Q4 (296.29-303.21) | 1.41 (1.25~1.59) | <0.001 | 1.34 (1.2~1.51) | <0.001 | 1.27 (1.13~1.42) | <0.001 | 1.26 (1.12~1.42) | <0.001 |
| Q5 >303.21 | 2.43 (2.18~2.71) | <0.001 | 2.13 (1.91~2.38) | <0.001 | 1.79 (1.59~2.00) | <0.001 | 1.79 (1.59~2.00) | <0.001 |
| P for trend |  | <0.001 |  | <0.001 |  | <0.001 |  | <0.001 |

HR, hazard ratio; CI, confidence interval; Ref, reference.

Crude model adjusted for none.

Model 1 adjusted for age, sex, ethnicity, height, and weight.

Model 2 adjusted for Model 1+heart rate, SPO2, temperature, SBP, DBP, UO, bicarbonate, calcium, WBC, PLT, hemoglobin, SCr, and PH.

Model 3 adjusted for Model 2+ AKI, diabetes mellitus, congestive heart failure, chronic pulmonary disease, ventilator use, and loop diuretics use.

**Table S3. Association of osmolality levels with 90-day mortality in patients with sepsis after combining Q2 and Q3.**

| **Variable** | **Crude Model** | | **Model 1** | | **Model 2** | | **Model 3** | |
| --- | --- | --- | --- | --- | --- | --- | --- | --- |
|  | **HR 95% CI** | ***P*-value** | **HR 95% CI** | ***P*-value** | **HR 95% CI** | ***P*-value** | **HR 95% CI** | ***P*-value** |
| Q1 ≤ 285.80 | 1.48 (1.34~1.63) | <0.001 | 1.53 (1.39~1.69) | <0.001 | 1.52 (1.37~1.67) | <0.001 | 1.50 (1.36~1.65) | <0.001 |
| Q2+Q3 (285.80-296.29) | 1(Ref) |  | 1(Ref) |  | 1(Ref) |  | 1(Ref) |  |
| Q4 (296.29-303.21) | 1.42 (1.28~1.56) | <0.001 | 1.33 (1.21~1.47) | <0.001 | 1.25 (1.13~1.38) | <0.001 | 1.25 (1.13~1.38) | <0.001 |
| Q5 >303.21 | 2.44 (2.23~2.66) | <0.001 | 2.12 (1.94~2.31) | <0.001 | 1.77 (1.61~1.94) | <0.001 | 1.77 (1.61~1.95) | <0.001 |
| Trend.test |  | <0.001 |  | <0.001 |  | <0.001 |  | <0.001 |

HR, hazard ratio; CI, confidence interval; Ref, reference.

Crude model adjusted for none.

Model 1 adjusted for age, sex, ethnicity, height, and weight.

Model 2 adjusted for Model 1+heart rate, SPO_2_, temperature, SBP, DBP, UO, bicarbonate, calcium, WBC, PLT, hemoglobin, SCr, and PH.

Model 3 adjusted for Model 2+ AKI, diabetes mellitus, congestive heart failure, chronic pulmonary disease, ventilator use, and loop diuretics use.

**Figure S1. Relationship between serum osmolality and 90-day mortality**

**Figure S2 Subgroup analysis of the association between 28-day mortality and osmolality.**

Stratified curve fitting based on sex (A), age (B), AKI(C), and Diabetes mellitus (D).

**Figure S3 Curve fitting between serum osmolality and risk of mortality based on patient source.**

Notes: A: Relationship between serum osmolality and 28-day mortality risk; Ｂ：Relationship between serum osmolality and 90-day mortality risk.

**Figure S4 Curve fitting between serum osmolality and risk of mortality based on septic shock.**

Notes: A: Relationship between serum osmolality and 28-day mortality risk; Ｂ：Relationship between serum osmolality and 90-day mortality risk.

**Table S4 Distribution of the amount of fluid received on the first day in each group.**

| **Variables** | **Total** | **Baseline serum osmolality levels (mmol/L)** | | | | | ***P*-value** |
| --- | --- | --- | --- | --- | --- | --- | --- |
|  |  | **Q1** | **Q2** | **Q3** | **Q4** | **Q5** |  |
| Input _1day_ (ml) | 3343.9 (2009.4, 5045.5) | 3360.0 (2005.6, 5029.1) | 3359.9 (2154.4, 4929.1) | 3383.4 (2208.3, 5038.4) | 3312.0 (1905.0, 5046.7) | 3288.7 (1812.1, 5181.7) | 0.242 |

**Figure S5 Curve fitting between serum osmolality and mortality risk in 3 subgroups based on fluid volume.**

Notes: A: Relationship between serum osmolality and 28-day mortality risk; Ｂ：Relationship between serum osmolality and 90-day mortality risk.

**Table S5 Distribution of infection sites in each group**

| **Variables** | **Total** | **Baseline serum osmolality levels (mmol/L)** | | | | | ***P*-value** |
| --- | --- | --- | --- | --- | --- | --- | --- |
|  |  | **Q1** | **Q2** | **Q3** | **Q4** | **Q5** |  |
| Infection site |  |  |  |  |  |  | < 0.001 |
| Lung (%) | 5758 (43.6) | 1155 (43.7) | 1096 (41.5) | 1080 (40.9) | 1196 (45.2) | 1231 (46.6) |  |
| Urinary (%) | 2633 (19.9) | 496 (18.8) | 453 (17.1) | 458 (17.3) | 532 (20.1) | 694 (26.2) |  |
| Other (%) | 4828 (36.5) | 993 (37.6) | 1093 (41.4) | 1104 (41.8) | 919 (34.7) | 719 (27.2) |  |

**Figure S6 Curve fitting between serum osmolality and risk of mortality based on site of infection.**

Notes: A: Relationship between serum osmolality and 28-day mortality risk; Ｂ：Relationship between serum osmolality and 90-day mortality risk.
